# Spatial and temporal characteristics of temperature effects on cardiovascular disease in Southern China using the Empirical Mode Decomposition method

**DOI:** 10.1038/s41598-018-33184-6

**Published:** 2018-10-03

**Authors:** Jixia Huang, Li Wang, Shibo Wang, Yaling Lu, Weiwei Zhang, Jinfeng Wang

**Affiliations:** 1 Key Laboratory for Silviculture and Conservation of Ministry of Education, Beijing Forestry University, Beijing, 100083 China; 2 The Key Laboratory of Land Surface Pattern and Simulation, Chinese Academy of Science, Beijing, 100101 China; 3 State Environmental Protection Key Laboratory of Environmental Planning and Policy Simulation, Chinese Academy for Environmental Planning, Beijing, 100012 China; 4 School of Environmental Science and Engineering, Suzhou University of Science and Technology, Suzhou, 215011 China; 5 State Key Laboratory of Resources and Environmental Information System, Chinese Academy of Sciences, Beijing, 100101 China

## Abstract

Until now, few studies have analyzed the effects of temperature on cardiovascular disease (CVD) death at different time points. In this study, we chose 9 different cities in the subtropical and tropical areas of China and analyzed the correlation between temperature at different time points and CVD mortality. We completed this study in two steps. First, we analyzed different time trend decomposition data related to CVD mortality in different populations within the 9 selected cities using empirical mode decomposition (EMD). Second, we created a regression fitting analysis of CVD mortality and temperatures at different time periods. The results showed that the CVD mortality of subtropical and tropical areas in southern Chinese cities represented spatial heterogeneity. The CVD mortality rates in Beihai, Hefei and Nanning showed rising trends, whereas the CVD mortality rates in Haikou, Guilin and Changde appeared to be decreasing. At the daily, seasonal and year time scales, low temperatures were negatively correlated with CVD mortality. Other than at the daily time scale, high temperatures did not significantly influence CVD mortality. This article will help to develop appropriate measures to reduce temperature-related mortality risk in different populations within the subtropical and tropical regions of China.

## Introduction

Within the past century, global temperatures in most areas have presented a warming trend^1^. In 70–75% of the world, including North America^2^, Central and Western Europe^3^ and China^4^, the increased numbers of warm nights have been very obvious. But some local areas, including North America, the eastern United States and southern Greenland, are experiencing more cold days and less warm days^5^.

The dramatic changes in global temperatures have had a certain negative impact on human health^6,7^.Cardiovascular disease (CVD) has a close relationship with extreme temperatures. CVD represents a major cause of death each year in China^8^. Therefore, it is very important to study temperature-related effects on Chinese CVD patients.

Previous studies have shown that extremely low temperatures induce a 1–2 week lag in CVD onset. However, extremely high temperatures are typically short in duration(1–2 days)^9^. The influence of extreme temperatures on different populations of patients with CVD varies^10^. Previous research has shown that extreme temperature exposure is more damaging to females and the elderly than to men and the young^11–13^.

Thus far, many studies regarding the influence of extreme temperatures on CVD deaths have focused on short-term effects and hysteresis^6,7^. Few studies focused on the long-term interactions between temperature exposure and CVD (e.g., exposure for months, quarters, or years). Existing studies have shown that the average temperature for a long period of time will also affect the death rate of a given population^14^. In different seasons, the mortality rate due to coronary heart disease also differs^13^. So far, researches regarding the long-term effects of temperature on disease has been studied in suicide and coronary heart disease^14^, ventricular arrhythmias^15^, CV-related hospitalizations^16^ and so on. However, the long-term effects of temperature on death due to CVD in China are still insufficient. Thus, this study targeted the long-term impacts of temperatures at different time point (moon, quarter, and year) on cardiovascular disease death in Chinese patients and sought to identify groups who are vulnerable to temperature-related effects.

## Data and Methods

**Figure 1:**
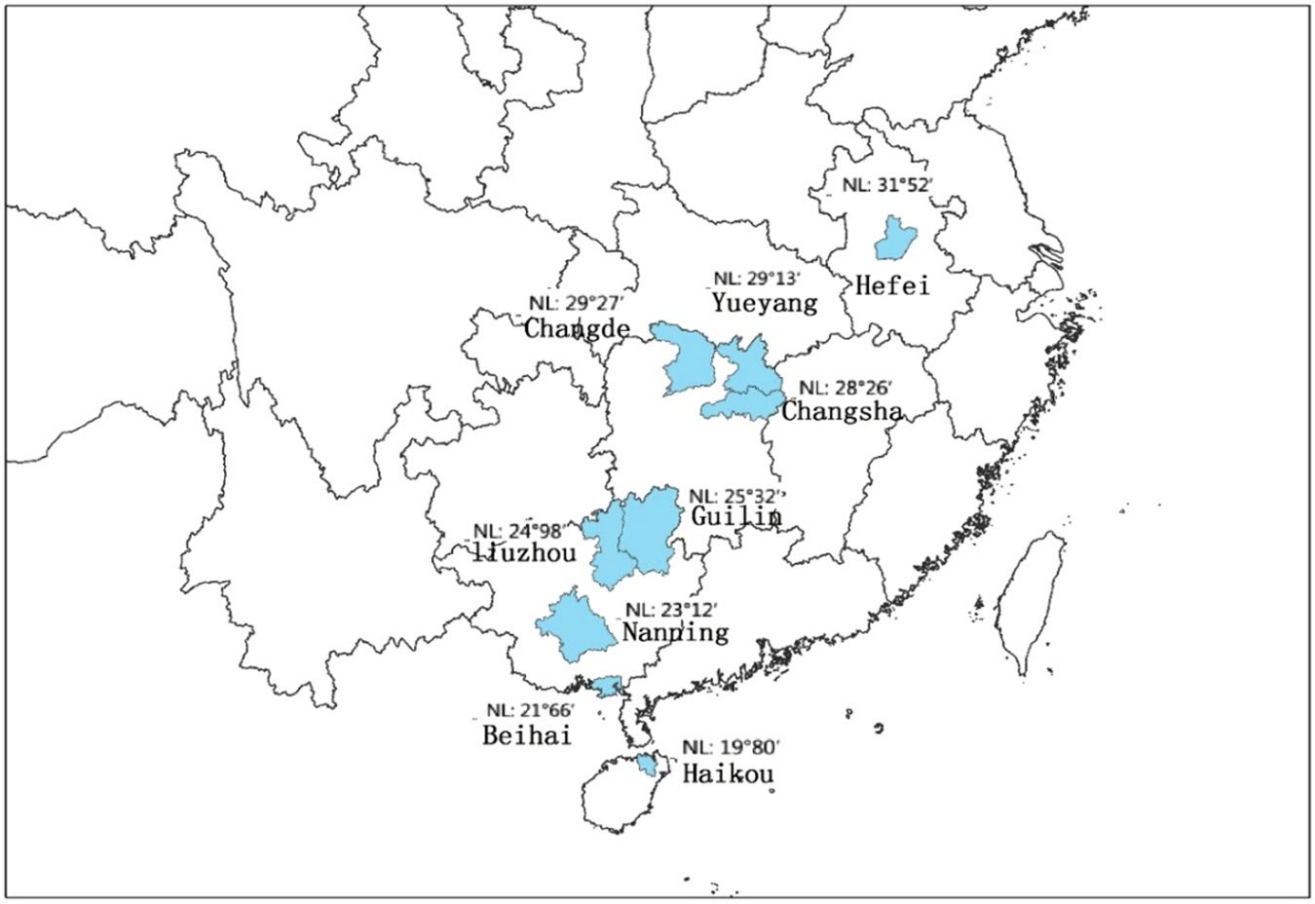
The geographical distribution of the nine research cities.

The death data in this study were collected from death surveillance of disease surveillance system in China, including gender, permanent address, primary cause of death, classification of primary cause of death, age and date of death. The causes of CVD were classified according to the International Classification of Disease 10th version (ICD-10:I00-I79). The death data were provided by the Chinese Center for Disease Control and Prevention. The data were obtained from nine death-monitoring sites in southern China between January 1, 2008 and December 31, 2011.These data included daily CVD death information over a total of 1461 days. The nine death-monitoring sites included: Hefei–Anhui Province, Changsha, Changde and Yueyang–Hunan Province, Nanning, Beihai, Guilin and Liuzhou–Guangxi Province, and Haikou–Hainan Province. Figure 1 below shows the spatial distribution of these nine cities. The CVD death population was assessed overall and divided into male and female subgroups as well as people over 65 years of age and people under 65 years of age.

Daily meteorological data (maximum, minimum, and mean temperatures and atmospheric pressure and relative humidity) for the same time period were collected from the China Meteorological Data Sharing Service System. Each city has two meteorological monitoring stations; the average of the meteorological data from both stations was used.

Based on World Health Organization and European Centre for Environment and Health methodology, air pollution indices (APIs) were calculated using respirable particulate matter (PM_10_), sulfur dioxide (SO_2_), and nitrogen dioxide (NO_2_) measurements obtained by the monitoring stations^17^. The API can replace detailed air pollution data (such as PM_10_ and SO_2_) and act as a comprehensive air quality indicator^18,19^. In this study, the APIs were obtained from the Chinese Environmental Protection Agency.

### Temporal period and trend decomposition

In this study, we used EMD to analyze the time series of death in each city. EMD is an adaptive time series data analysis model proposed by Huang *et al*.^20,21^. In EMD analysis, the original data are decomposed into a series of modes without requiring prior knowledge. Compared with Fourier and wavelet decomposition, EMD has its own advantages. The Fourier analyses can transform data into a combination of sine and cosine functions with different frequencies, whereas wavelet analysis requires wavelet splines. These types of decomposition involve many spurious components due to serious restriction of the harmonic nature of the basis function^19^. The EMD method decomposes the data into several oscillatory components, which is called the eigenfunction, corresponding to some physical phenomenon underlying the data, and the residual of the decomposition represents the trend of the data^19^. The detailed description of EMD was provided in the supplementary appendix.

The values of CVD mortality in different time scales (the eigenfunction: IMF_1_-IMF_n_) were obtained by EMD decomposition. In this study, we hoped to disclose the effects of low temperature and high temperature on CVD death at different time scales. The time points were divided into two periods. April–September were designated as the warm months, and October–March as the cold months.

According to the cycle length of the eigenfunction, the values of days (i.e., original), week, month, season and year were selected. We used poisson regression analysis to analyze the relationship between CVD mortality and meteorological factors in cold and warm months under different time scales. The meteorological factors includes mean temperature, daily temperature range, air pressure, relative humidity, rainfall, air pollution index.

## Results

### Overview of the dataset

Overall, the distribution of meteorological factors and APIs in these nine cities are significantly different. With the decrease of latitude, the daily average temperature increased gradually from 16.4 °C in Hefei to 23.9 °C in Haikou. The daily temperature range went through a process from small to large and then smaller. From 7.9 °C in Hefei to 9.5 °C in Yueyang City, then gradually decreased to 6.3 °C in Haikou City. The trend of wind speed was just the opposite, going from large to small and then larger. The average daily wind speed in Hefei decreased from 2.4 m/s to 1.2 m/s in Yueyang City, and then gradually increased to 3.9 m/s in Haikou City. The relative humidity and rainfall increased with the increase of latitude. In terms of average APIs, Hefei was the most serious city, reaching 83.5, followed by Changde City, reaching 74.5, Liuzhou was the lightest, only 34.2.

The average daily CVD mortality rates in these nine cities were not significantly different, but the maximum daily CVD mortality rates were quite different. The difference between men and women was not large, but the average daily mortality rate in individuals over the age of 65 was much greater than in those under the age of 65 (Table 1).

**Table 1:** The CVD daily mortality among all ages, males, females, those greater than 65 years of age, and those younger than 65 years of age in the nine cities (/100,000 persons).

City	Overall	Male	Female	>=65	<65
Hefei	0.8	0.4	0.4	0.7	0.1
Changsha	1.0	0.6	0.4	0.9	0.1
Changde	0.5	0.3	0.2	0.4	0.1
Yueyang	1.0	0.6	0.4	0.8	0.2
Nanning	1.1	0.6	0.5	0.9	0.2
Liuzhou	0.5	0.3	0.2	0.4	0.1
Guilin	0.7	0.4	0.3	0.5	0.1
Beihai	0.8	0.5	0.3	0.7	0.1
Haikou	0.5	0.3	0.2	0.4	0.1

For all population types, the average daily mortality rate was between 0.5–1.1 per 100,000 people. Yueyang City’s daily mortality rate was the highest and reached 9.6/100,000 people, whereas other cities had highest daily mortality rates of 2–3 people/10 million people. The average daily mortality rates of Changsha, Yueyang and Nanning were high and were closer to more than 1 person/100,000 people. For men, the average daily mortality rate was between 0.3–0.6 per 100,000 people. Yueyang City had the highest mortality rate of up to 4.7 people/10 million people. For women, the average daily mortality rate was between 0.2–0.4/100,000 people, which was slightly less than the mortality rate observed for men. The average mortality rate among adults over the age of 65was between 0.4–0.9/100,000, and the average daily mortality rate among people under 65 was between 0.1–0.2/100,000. The difference between these two mortality rates was nearly four-fold (Table 1).

### Daily CVD mortality rate time period decomposition in the nine cities

**Figure 2:**
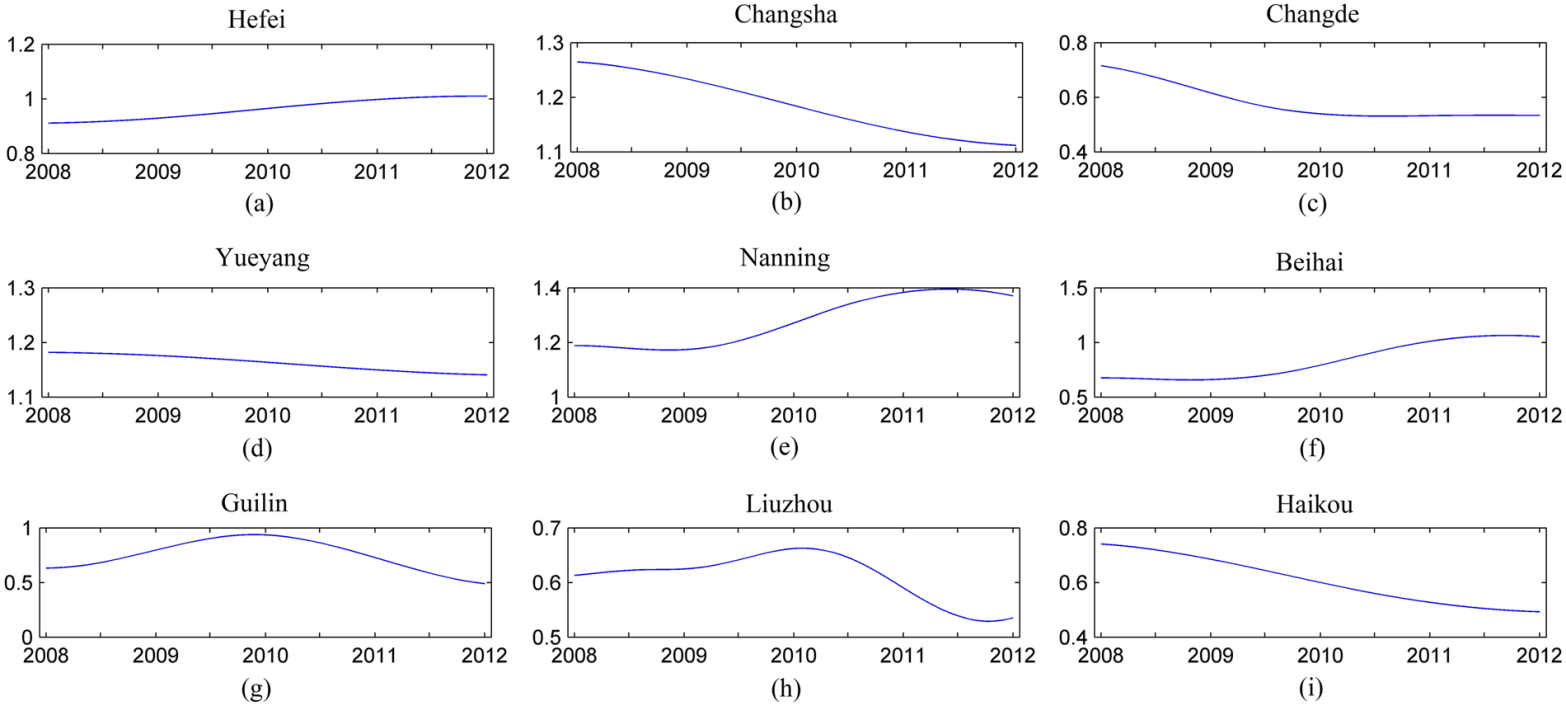
The EMD decomposition results of CVD mortality for all populations in 9 cities during the cold months (October–March).

We used the EMD method to analyze trends in CVD mortality among different populations in these nine cities. Figure 2 below shows the trends in CVD deaths for all populations of all nine cities during the cold months. In each plot, the subgraph represents the long-term trend of the variable. The average daily mortality rate in Changsha, Changde, Yueyang, Liuzhou, Guilin and Haikou during the cold months declined, whereas the average daily mortality rate in Hefei, Nanning and Beihai increased.

The death rate trends during the warm months varied across the cities. In Hefei, Changsha City and Beihai City, the overall average daily mortality rates increased. Hefei’s average daily mortality rate increased from 0.7/10 million to 0.8/10 million people, in Changsha the daily mortality rate increased from 0.85/10 million to 0.95/10 million people, and in Beihai, the daily mortality rate increased from 0.5/10 million people to 0.8/10 million or so. In Yueyang and Liuzhou, a significant downward trend appeared. Yueyang’s daily mortality rate decreased from 0.9/10 million to 0.7/10 million people. The trends in several other cities were not obvious and typically fluctuated.

### Daily CVD death rate trends in different populations during cold months

The daily CVD death rates in nine cities during the cold months was as shown in Table 2. The CVD mortality rates in Nanning, Changsha, Yueyang and Hefei exceeded 0.7/10 million, and Haikou had the lowest mortality rate. The mortality rate of men is generally higher than women, and that of the elderly is higher than young people.

**Table 2:** The CVD daily mortality among all ages, males, females, those greater than 65 years of age, and those younger than 65 years of age in 9 cities during the cold months (/100,000 persons).

City	Overall	Male	Female	>=65	<65
Hefei	0.73	0.38	0.35	0.64	0.09
Changsha	0.87	0.48	0.38	0.72	0.15
Changde	0.44	0.24	0.19	0.35	0.08
Yueyang	0.78	0.45	0.33	0.63	0.15
Nanning	0.91	0.51	0.39	0.73	0.18
Liuzhou	0.44	0.26	0.19	0.33	0.12
Guilin	0.51	0.32	0.19	0.45	0.06
Beihai	0.56	0.33	0.23	0.49	0.07
Haikou	0.37	0.21	0.16	0.29	0.09

The changes in CVD mortality in nine cities during the cold month are shown in Table 3. In general, the mortality rate of CVD in these nine cities showed obvious spatial heterogeneity in cold months. The mortality rate of CVD in different populations of Hefei, Nanning and Beihai was mainly increased. While, the mortality rates in Changsha, Changde, Liuzhou, Guilin and Haikou decreased. Among them, the death rate in Beihai City rose fastest, reaching 0.41/10 million.

**Table 3:** The change trend in CVD daily mortality from 2008–2011 during the cold months (October–March).

City	Overall	Male	Female	>=65	<65
Hefei	+0.10↑	—	+0.02↑	+0.12↑	−0.05↓
Changsha	− 0.15↓	−0.05↓	—	+0.01↑	−0.03↓
Changde	− 0.18↓	—	− 0.05↓	−0.11↓	—
Yueyang	− 0.05↓	+0.05↓	− 0.08↓	+0.04↑	—
Nanning	+0.22↑	+0.23↑	+0.17↑	+0.28↑	—
Liuzhou	—	−0.07↓	−0.03↓	+0.01↑	−0.03↓
Guilin	—	−0.21↓	−0.10↓	—	—
Beihai	+0.41↑	+0.34↑	+0.05↑	+0.32↑	+0.03↑
Haikou	−0.25↓	−0.27↓	−0.05↓	−0.40↓	−0.08↓

### Death rate trends for different populations during the warm months

The daily CVD death rates in nine cities during the warm months as shown in Table 4. Compared with the table, the CVD mortality rates in warm month were lower than that of cold month. The mortality rates in Nanning and Changsha exceeded 0.6/10 million, and the daily mortality in Liuzhou was the lowest.

**Table 4:** The CVD daily mortality among all ages, males, females, those greater than 65 years of age, and those younger than 65 years of age in 9 cities during the warm months (/100,000 persons).

City	Overall	Male	Female	>=65	<65
Hefei	0.58	0.29	0.28	0.49	0.09
Changsha	0.65	0.36	0.28	0.53	0.12
Changde	0.37	0.21	0.16	0.29	0.08
Yueyang	0.57	0.33	0.24	0.45	0.12
Nanning	0.70	0.38	0.32	0.56	0.14
Liuzhou	0.35	0.19	0.16	0.28	0.07
Guilin	0.38	0.19	0.19	0.31	0.31
Beihai	0.48	0.30	0.18	0.41	0.07
Haikou	0.37	0.21	0.16	0.29	0.08

The changes in CVD mortality in nine cities during the warm month are shown in Table 5. Overall, the mortality of CVD in the warm months in these nine cities showed obvious spatial heterogeneity. The mortality rate of CVD in Hefei, Changsha, Nanning and Beihai increased significantly in different population groups. While the mortality rates in Changde, Yueyang and Haikou decreased. The death rate in Beihai was the fastest, reaching 0.32/10 million.

**Table 5:** The change trends in CVD daily mortality from 2008–2011 during the hot months (April–September).

City	Overall	Male	Female	>=65	<65
Hefei	+0.11↑	+0.05↑	+0.04↑	+0.03↑	+0.01↑
Changsha	+0.05↑	—	—	+0.01↑	—
Changde	−0.20↓	−0.02↓	−0.08↓	−0.03↓	—
Yueyang	−0.22↓	—	−0.09↓	−0.19↓	−0.07↓
Nanning	—	+0.05↑	+0.03↑	—	+0.05↑
Liuzhou	—	−0.01↓	−0.11↓	+0.01↑	+0.04↑
Guilin	—	+0.12↑	+0.06↑	—	−0.09↓
Beihai	+0.32↑	+0.15↑	+0.17↑	+0.15↑	+0.02↑
Haikou	—	−0.08↓	—	−0.08↓	−0.05↓

### Multi scale Time Series Analysis of temperature effects on CVD Death

Tables 6–9 below show the results of different weather factors on CVD day mortality at different time scales during the cold months. Different time scales include days, months, seasons and years. (On the week scale, the regression equation in all cities did not pass the significance test, so it is not listed here. Blank values listed in the table indicate that the multiple regression coefficient didn’t through t test.) At different time scales, temperature exposure had different effects on CVD deaths. A negative correlation between low temperatures and CVD mortality rate at the time scale of the day (Table 6), season (Table 8) and year (Table 9) was observed. On the week and month scales, the correlations between temperature and the CVD death rate were not clear (Table 7). On the day scale, a negative correlation was observed between these nine cities’ low temperatures and their CVD mortality rates. The coefficient values for temperature in all cities were negative, of which Changsha and Beihai had the greatest absolute correlation coefficients at −0.05 and −0.04, respectively. These results indicated that the daily average temperature drop of 1 °C increased the average death rate by 0.05 and 0.04 per 10 million people. This demonstrated that on the day time scale, the lower the temperature, the higher the CVD mortality rate. At the season time scale, there was a negative correlation between the mean temperature of 7 cities and CVD mortality (Table 8). Changsha had the largest negative correlation of −0.03, whereas that of other cities was −0.01. At the year time scale, the correlation coefficient between mean temperature and CVD death was small, but in 7 cities, the correlations between the mean temperature and CVD mortality were negative (Table 9).

**Table 6:** The day scale effect of cold temperatures and CVD mortality for all ages.

City	AT	Trange	AP	RH	API	F
Hefei	−0.02		−0.001			14.42
Changsha	−0.05	0.01	−0.014		0.002	60.82
Changde	−0.01			−0.002	0.001	11.01
Yueyang	−0.01					18.69
Nanning	−0.03		−0.001			17.98
Beihai	−0.01				0.001	23.51
Haikou	−0.03	0.02	−0.002			9.50
Hefei	−0.04		−0.002		0.003	26.39
Changsha	−0.03	0.02	−0.002		0.004	8.01

**Table 7:** The month scale effect of cold temperatures and CVD mortality for all ages.

City	AT	Trange	AP	RH	API	F
Hefei	0.002	0.003				5.95
Nanning	−0.005					37.43
Liuzhou				−0.001		10.35
Guilin	−0.003	0.003				8.17
Beihai		0.004				4.20

**Table 8:** The season scale effect of cold temperatures and CVD mortality for all ages.

City	AT	Trange	AP	RH	API	F
Hefei		0.001				4.11
Changsha	−0.03		−0.008		0.002	90.99
Changde	−0.01			−0.002		42.96
Yueyang	−0.01		−0.001	−0.001		26.62
Nanning	−0.01					93.89
Beihai	−0.01			−0.001		43.77
Haikou	−0.01					131.7
Hefei	−0.01	0.003		0.001	0.001	19.12

**Table 9:** The year scale effect of cold temperatures and CVD mortality for all ages.

City	AT	Trange	Wind	RH	API	F
Hefei	−0.001		0.005	0.001		8.10
Changsha	−0.001	0.001				5.38
Changde	−0.007					63.16
Yueyang	−0.004					14.28
Nanning	−0.016		0.032		0.001	53.28
Beihai	−0.003		0.006		0.001	14.52
Haikou	−0.011				0.002	19.98

(AT: mean temperature (°C); Trange: daily temperature range (°C); AP: air pressure (Pa); RH: relative humidity (%); API: air pollution index; F: F value of model. The significance levers of all the coefficients listed in this Tables 6–9 <0.05. The coefficients in the table represent the number of CVD changes per 10 million people when the environmental factors change by one unit).

For the elderly, the effect of low temperature on CVD mortality also varied at different time scales. On the daytime scale, the average temperature was negatively correlated with CVD mortality in all cities, especially in Changsha and Beihai where the coefficient values were all −0.04. On the week and month time scales, the effect of hypothermia on CVD mortality was not very obvious. On the seasonal scale, six cities had negative correlations between temperature and CVD mortality. On the year scale, five cities had negative correlations between temperature CVD mortality.

We further analyzed the warm months and the average temperature influence (April–September) on different time scales on CVD mortality in all groups and elderly people. It was found that most of the effects were not significant except in a few cities where the temperature had a positive effect on CVD mortality.

## Discussion

Currently in Europe, the United States and some parts of China, researchers have studied the effects of extreme temperatures on CVD mortality within these populations^11,22–24^. However, these studies have mainly focused on the temperature threshold effect and short-term hysteresis^25–27^. Studies examining the effects of temperature on CVD mortality over different periods of time are still lacking. This study is the first to apply a quantitative approach in studying the relationship between air temperature and CVD mortality at different time scales in Chinese cities.

Low temperatures affect the body’s circulatory system. Blood supplied to the skin decreases when exposed to cold air, which results in an accumulation of blood in central organs, and the excess blood is then disposed of by removing salt and water. Some blood is removed by the kidneys as urine while some settles in general intercellular spaces^26,28^. High temperatures increase the density of the blood. In hot environments, blood vessels in the skin will expand in order to maintain body temperature, resulting in sweat expelling from the body. This decrease the salt and water in the body, increasing blood density and, consequently, its propensity for clotting^26,28^.

The results showed a spatial heterogeneity in the trends in CVD mortality in the subtropical and tropical regions of southern China, and the temperature-related effects on CVD death also differed over various time periods. Overall, CVD mortality rate in Beihai, Hefei and Nanning increased, whereas in Haikou, Guilin and Changde, the death rate decreased. The average daily mortality rate for elderly persons over the age of 65 was approximately four times that of people under the age of 65, whereas women had a slightly higher average daily mortality than did men. This may be because females have a higher risk for arrhythmia, ischemia, and high blood pressure, all of which are more affected by extreme hot and cold temperatures^13^. There was a negative correlation between the low temperature effects and CVD mortality on the day, season and year scales, and the relationship between temperature and CVD mortality was unclear on the week and month scales. The effect of high temperature on CVD mortality was not significant on the time scales except for the day scale. Previous studies have shown that the effect of low temperature on CVD death has a lag period of 1–2 weeks, and the risk effect has a tendency that rises first and then decreases. In addition, the effects of high temperature on CVD death also have a short lag (1–3 days)^28^. The results showed that the high temperature’s effect is limited to the day scale, and the low temperature has a negative effect on the day, season and year scales. Meanwhile, in terms of different crowds, no matter high or low the temperature, the impact on old individuals was the greatest. The CVD death trends in the region showed a strong spatial heterogeneity. At the city level in Beihai and Hefei, in the cold months and the warm months, the CVD mortality rate significantly increased. In Haikou City, the CVD mortality rate decreased. Beihai City had the most severe situation. In the cold months, the death reached up to 0.41/10 million people, whereas in the warm months, it increased to 0.32/10 million people. In the cold and warm months in Hefei, the CVD mortality rate increased to 0.1/10 million people. However, in the cold months, Haikou’s CVD mortality fell to 0.25/10 million people. There are many factors that could affect CVD mortality, such as the socioeconomic level of the region, the physical condition of the individual and the climate^28^. High and low temperatures affect different cardiovascular diseases. Generally, low temperatures will trigger myocardial ischemia and acute myocardial infarction^29,30^, while high temperatures will trigger the congestive heart failure. High temperatures will directly lead to CVD death while cold temperatures only have indirect effects^27^. This may explain why the lag periods are different for hot and cold temperatures.

In terms of climate, since Beihai is close to the sea, its humidity is relatively high. Although Haikou is also a coastal city, its temperature is 2–3 °C higher than that of Beihai. The combination of air temperature and air humidity may have a greater impact on CVD death. This may partly explain the difference between Beihai’s CVD death and Haikou’s CVD death.

However, different populations’ CVD mortality trends were somewhat similar. In most cities, there was an upward trend in one population’s CVD mortality, and the CVD mortality rates of other populations also increased. People over the age of 65 had the greatest changes in their CVD trends, especially during the cold months. Whether growing or declining, the changes were very significant. The changing trends in CVD death rates in Beihai and Nanning were the most significant, and during the cold period, the rates increased to 0.32/10 million and 0.28/10 million people, respectively. However, the rate in Haikou City declined to 0.4/10 million people. This suggested that the changes in CVD were more obvious for older individuals, whereas those in the younger population were not significant. Therefore, when cold or heat waves come, we need to pay more attention to the elderly.

The low temperature had a negative effect on CVD mortality on the day, season, and year scales. But on the week and month scales, the relationship between low temperature and CVD mortality was unclear. The result also showed that among cities, low temperatures had different impacts on different people at different time scales. This may be due to variable climates, socioeconomic levels, medical facilities and other factors that modulate different effects. Many studies have confirmed that one or a few days of low temperatures can increase the CVD mortality rate^31^. However, studies about the relationship between hypothermia and CVD mortality at the time and season scales are still needed. The results revealed that not only short-term low-temperature exposure can increase the risk of CVD death, but so too can medium- and long-term exposure. Therefore, when the seasonal average temperature or the average annual temperature decreases, the risk of CVD death increases.

The results showed that high temperatures have a significant effect on CVD mortality at daytime scales, and the correlation was not statistically relevant at other time scales. In different cities, the impact of high temperatures on CVD mortality also varied. Previous studies have shown that high temperatures have a short periodicity on CVD mortality, which is generally 1–3 days^28,31^. The results from this study further confirmed this conclusion. On the other hand, this study also demonstrated that the impact of high temperatures is shorter when they last more than 1 week. However, the impact on CVD death is less. This paper further revealed the spatial heterogeneity of the high temperature effects on CVD death, indicating that in different cities, it is necessary to take corresponding measures to prevent heat wave weather.

The limitations to this study should be considered. First, the four years of data for the nine cities may not be sufficient. In future studies, our target is to collect more long-term data. Second, as an ecologic study, the temperature-related CVD mortality focused on population-level exposure, and the conclusion may not be applied at the individual level. Third, detailed air pollution data (such as PM_10_, SO_2_, and NO_2_) were not available, and APIs were therefore used instead; however, this method has been successfully used in previous research^17^.

## Conclusion

The results of this study reveal that on one hand, in southern China, different cities have variable CVD mortality trends and increases in CVD mortality rates mainly occurred in the elderly population. On the other hand, this study also showed that temperature effects on the population are different over different periods of time. Low temperatures can have an effect on CVD death at the day, season, and year scales, whereas high temperature only affects CVD mortality at the day scale. There are two major results of this study: First, considering that the impact of temperature on CVD patients has spatial heterogeneity, a differentiated extreme temperature led-CVD death warning system should be established in different regions. Second, the mean global temperature, with increased record numbers of warm days and heat waves, has been rising in recent years. Thus, the government should pay more attention to heat-related CVD mortality issues.

## Data Availability

Te datasets generated during and/or analysed during the current study are available from the corresponding author on reasonable request.
